# Spinal Hemangioblastoma: The Role of Imaging Characteristics in Preoperative Diagnosis and Surgical Planning

**DOI:** 10.7759/cureus.82740

**Published:** 2025-04-21

**Authors:** Zheting Zhang, Shiong Wen Low, Ira Sun, Su Lone Lim, Char Loo Tan, Chun Peng Goh

**Affiliations:** 1 Medicine, Lee Kong Chian School of Medicine, Nanyang Technological University, Singapore, SGP; 2 Neurosurgery, Ng Teng Fong General Hospital, Singapore, SGP; 3 Pathology, National University Hospital, Singapore, SGP

**Keywords:** intramedullary spinal cord tumors, magnetic resonance imaging, neurosurgery, spinal cord neoplasms, spinal hemangioblastoma, spine surgery

## Abstract

Spinal hemangioblastomas are rare, highly vascular tumors of the central nervous system (CNS) that may occur sporadically or in association with von Hippel-Lindau disease. These tumors pose significant diagnostic and surgical challenges due to their intramedullary location and propensity for hemorrhage. Given their radiographic overlap with other spinal cord tumors, imaging plays a crucial role in preoperative diagnosis and surgical planning. We present the case of a 58-year-old woman who developed progressive left lower limb weakness and sensory impairment over two years. Magnetic resonance imaging (MRI) revealed a lobulated, heterogeneously enhancing solid-cystic lesion within the spinal canal at the T11-12 level, with a diffuse syrinx spanning the entire cord. Surgical resection was performed, with a highly vascular lesion encountered intraoperatively. Histopathology confirmed the diagnosis of hemangioblastoma, classified as World Health Organization (WHO) grade 1. Reviewing the literature, certain radiographic features can aid in the diagnosis of spinal hemangioblastomas, improving preoperative planning for patients with these tumors. Surgical implications include the contraindication to biopsy due to the tumor’s vascularity, as well as preparation of blood products and preoperative patient counseling.

## Introduction

Intramedullary spinal cord tumors are rare, constituting 4-10% of all central nervous system (CNS) tumors and 20-30% of all intraspinal tumors [1-3]. Among intramedullary tumors, ependymomas and astrocytomas are the most common, accounting for more than 90% [2,4], followed by hemangioblastomas. Spinal hemangioblastomas are rare tumors accounting for 1.6-5.8% of all spinal cord tumors and 1-2.5% of all tumors of the CNS [2,5-7]. Approximately 75% of hemangioblastomas occur sporadically, while 25% are associated with von Hippel-Lindau disease and may be multiple in occurrence [6]. Spinal hemangioblastomas are benign, slow-growing tumors classified as World Health Organization (WHO) grade 1 in the 2021 WHO classification of CNS tumors [8]. Despite their benign histology, spinal hemangioblastomas can cause significant neurological impairment, often secondary to related edema, cyst, and syrinx [9]. The most common locations are the cervical or thoracic cord [2,7]. Microsurgical resection is the primary treatment modality [9-11], with stereotactic radiosurgery as an option in select cases [12]. Given their radiographic overlap with other intramedullary lesions such as ependymomas, astrocytomas, and cavernous malformations, precise imaging characterization is critical for preoperative planning. Magnetic resonance imaging (MRI) is the imaging modality of choice for the identification and characterization of spinal hemangioblastomas [13]. On contrast-enhanced T1-weighted MRI, the tumors appear as bright enhancing lesions, while T2-weighted MRI can better define the lesion and show associated edema or syrinx [14-16]. Although the utility of MRI in diagnosing spinal hemangioblastomas is well-recognized, the imaging features of certain cases may not always be distinct, posing challenges for clinical diagnosis and treatment [17]. Here, we present a case of a thoracic spinal hemangioblastoma and review the literature on the radiographic features of spinal hemangioblastoma, which may allow preoperative recognition and consequent surgical planning.

## Case presentation

We present the case of a 58-year-old woman with no significant medical history who developed progressive left lower limb weakness and sensory loss over two years. She experienced an episode of left knee giving way while walking, followed by worsening weakness that eventually required a walking stick for ambulation. Neurological examination revealed a left foot drop, with ankle dorsiflexion and hallux extension graded 1/5. There was patchy sensory loss in the left lower limb, while anal tone and perianal sensation remained intact.

**Figure 1:**
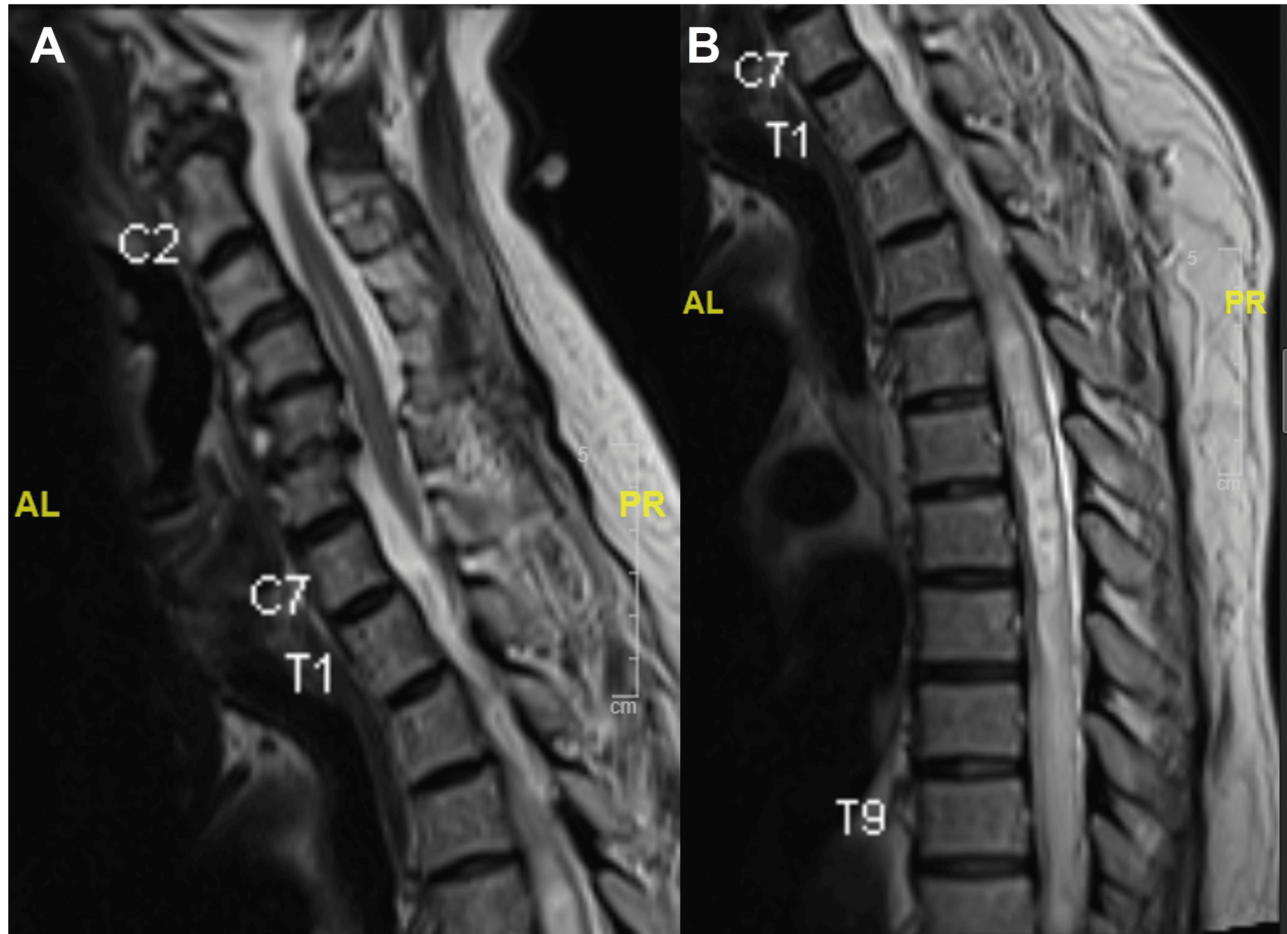
T2-weighted imaging of the MRI whole spine demonstrating diffuse syrinx along the entire spinal cord with associated cord expansion

**Figure 2:**
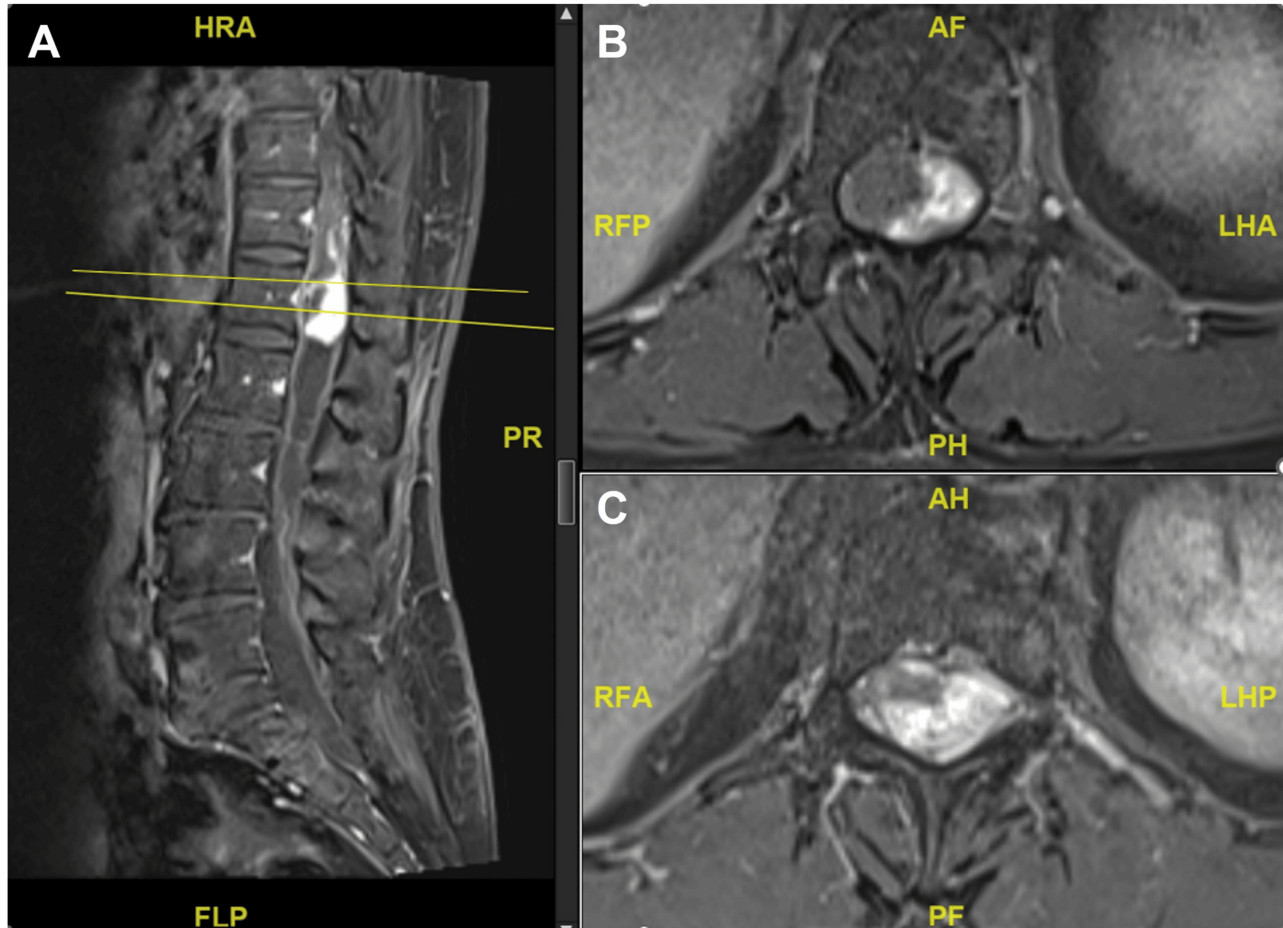
T1-weighted imaging with contrast MRI of the lumbar spine revealed a lobulated heterogeneously enhancing solid-cystic lesion within the spinal canal at the T11-12 levels.

MRI of the whole spine demonstrated a diffuse syrinx spanning the entire spinal cord with associated cord expansion. A lobulated, heterogeneously enhancing solid-cystic lesion was identified within the spinal canal at the T11-12 level (Figures 1, 2).

**Figure 3:**
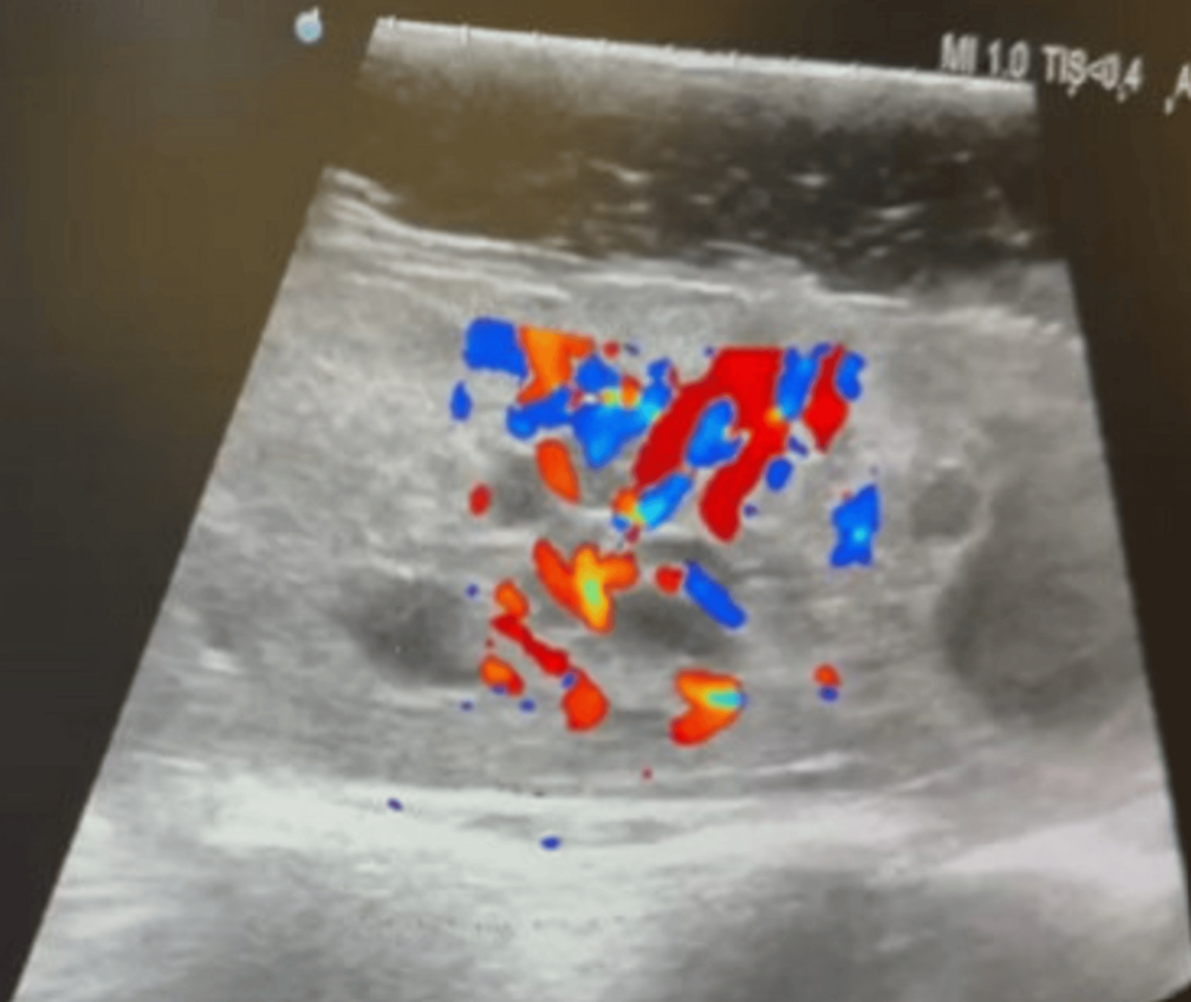
Intraoperative Doppler ultrasound at the surface of the dura revealing a highly vascular tumor

**Figure 4:**
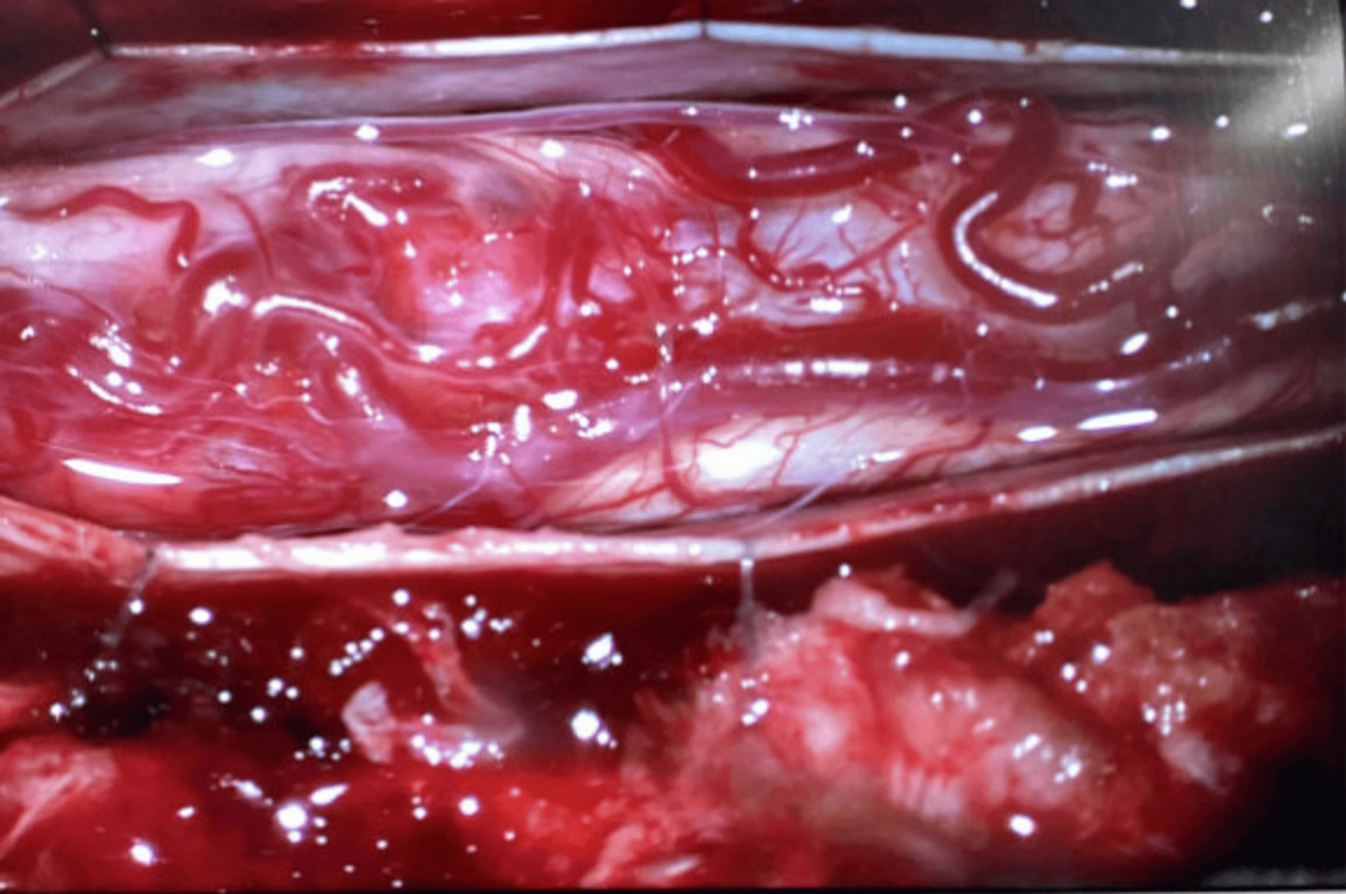
Engorged venous complex encountered at the surface of the spinal cord after durotomy

**Figure 5:**
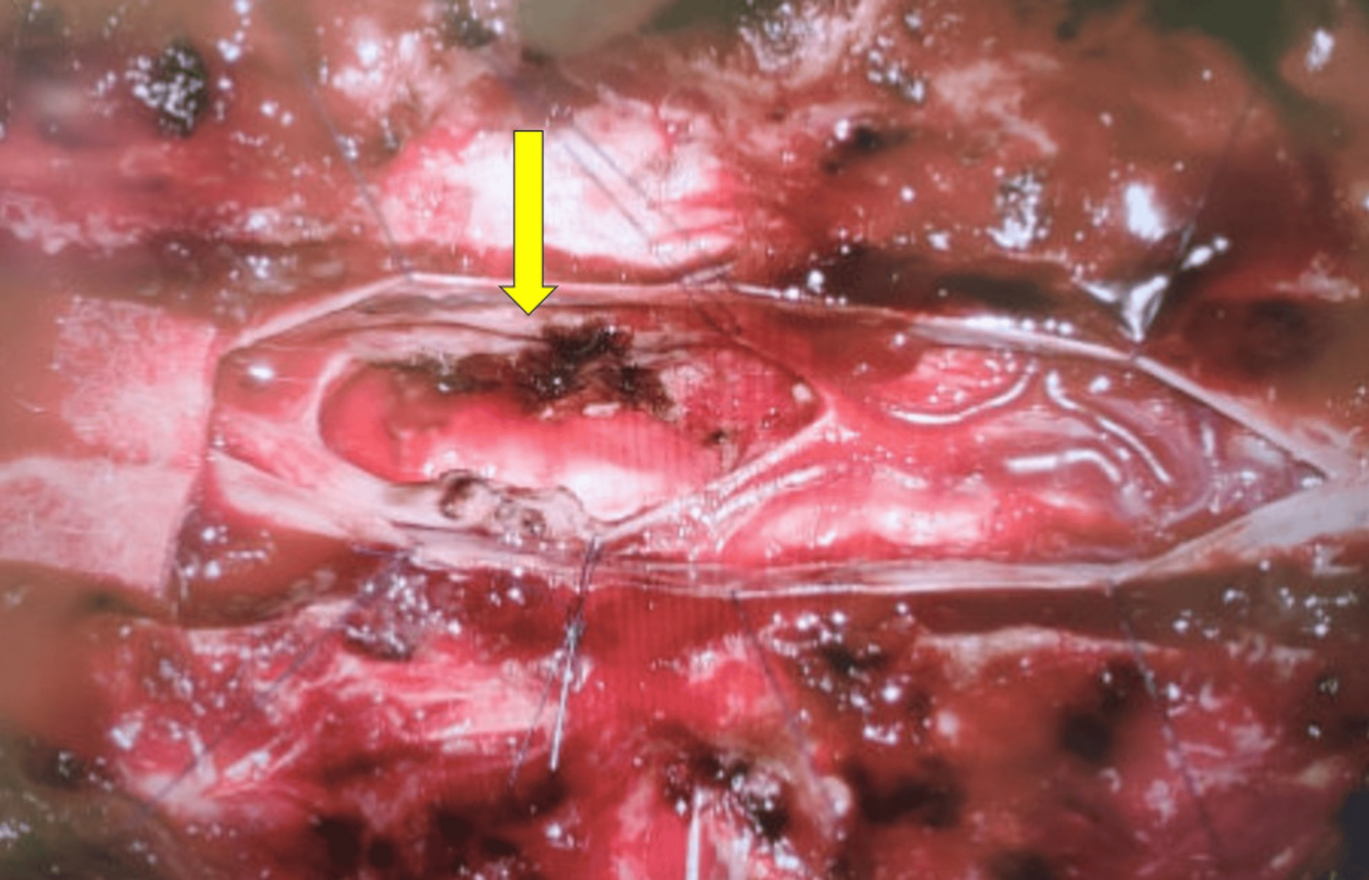
Surgical cavity at the end of the surgery The yellow arrow depicts the area of tumoral bleed requiring hemostasis with bipolar diathermy, and resection was limited due to intraoperative neuromonitoring signal drop.

**Figure 6:**
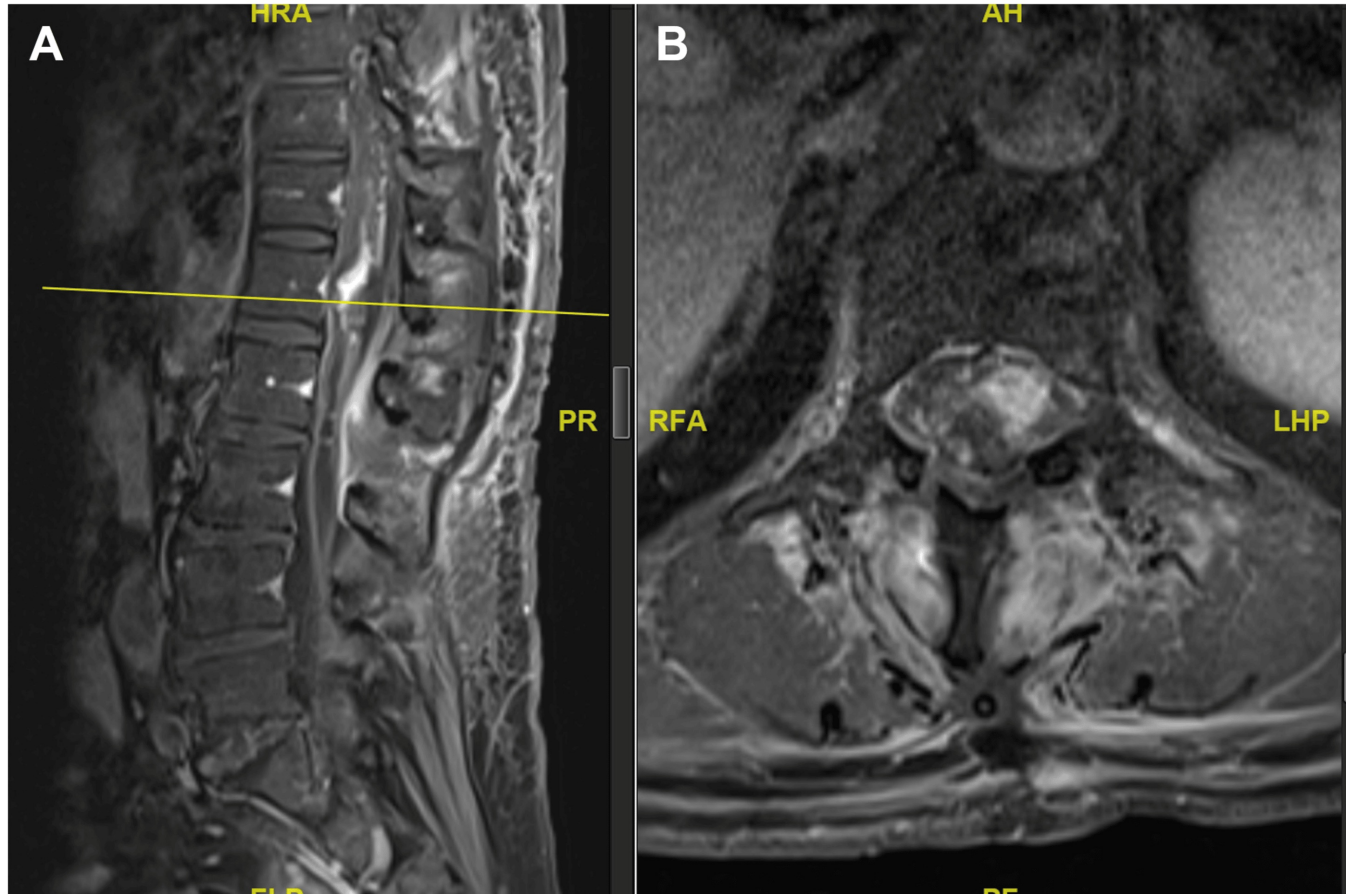
Postoperative day one: MRI of the T1-weighted imaging showing a residual tumor at the T11-12 level

The patient underwent posterior approach tumor resection with intraoperative neuromonitoring. Doppler ultrasound at the dural surface identified a highly vascular lesion (Figure 3). Upon durotomy, an engorged venous complex was encountered at the spinal cord surface (Figure 4). The tumor had presented itself on the left dorsolateral pial surface. It had a discernible tumor-cord interface at the superior and inferior poles, both located within the syrinx, as well as the anterior and right aspects. However, a residual tumor was left at the left lateral aspect due to neuromonitoring signal deterioration (Figure 5). Postoperative MRI confirmed the presence of residual tumor (Figure 6).

**Figure 7:**
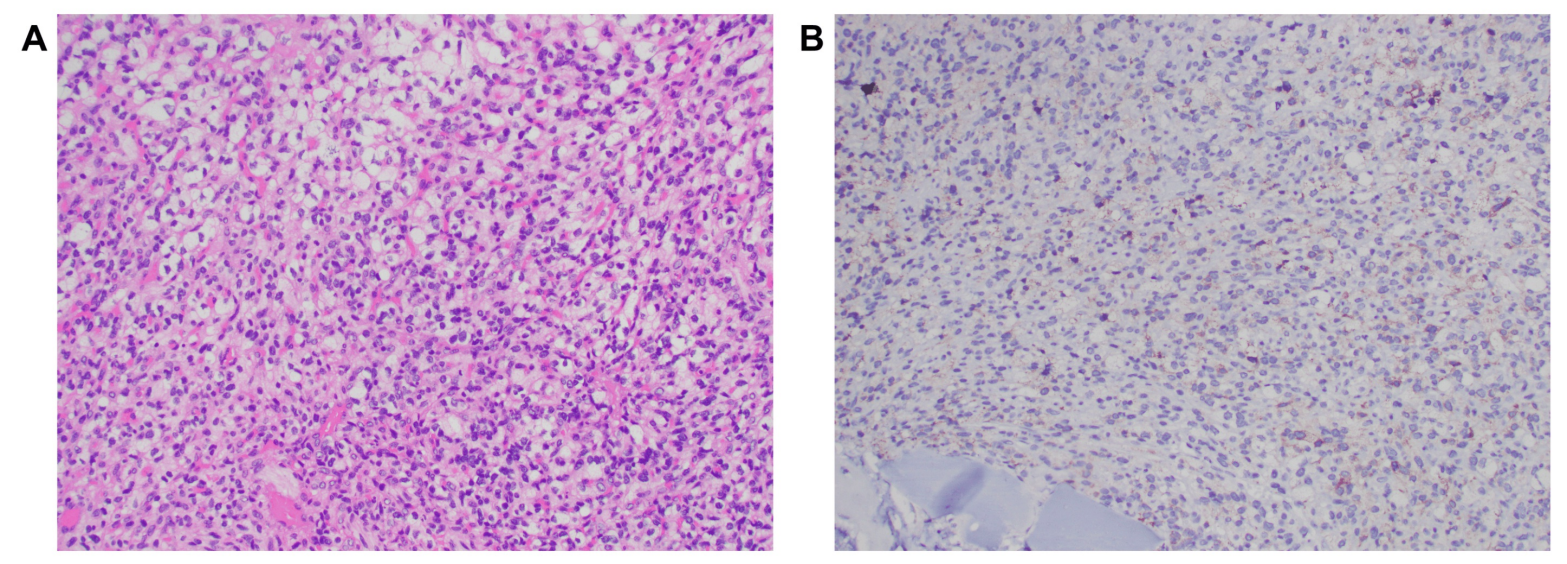
Histological examination (A) A tumor composed of stromal cells with some nuclear pleomorphism and clear cytoplasm, set in a rich capillary network. (B) The tumor cells were focally positive for alpha-inhibin.

Histopathology confirmed the diagnosis of hemangioblastoma, classified as CNS WHO grade 1 (Figure 7). MRI of the brain and CT of the abdomen and pelvis showed no evidence of von Hippel-Lindau syndrome.

**Figure 8:**
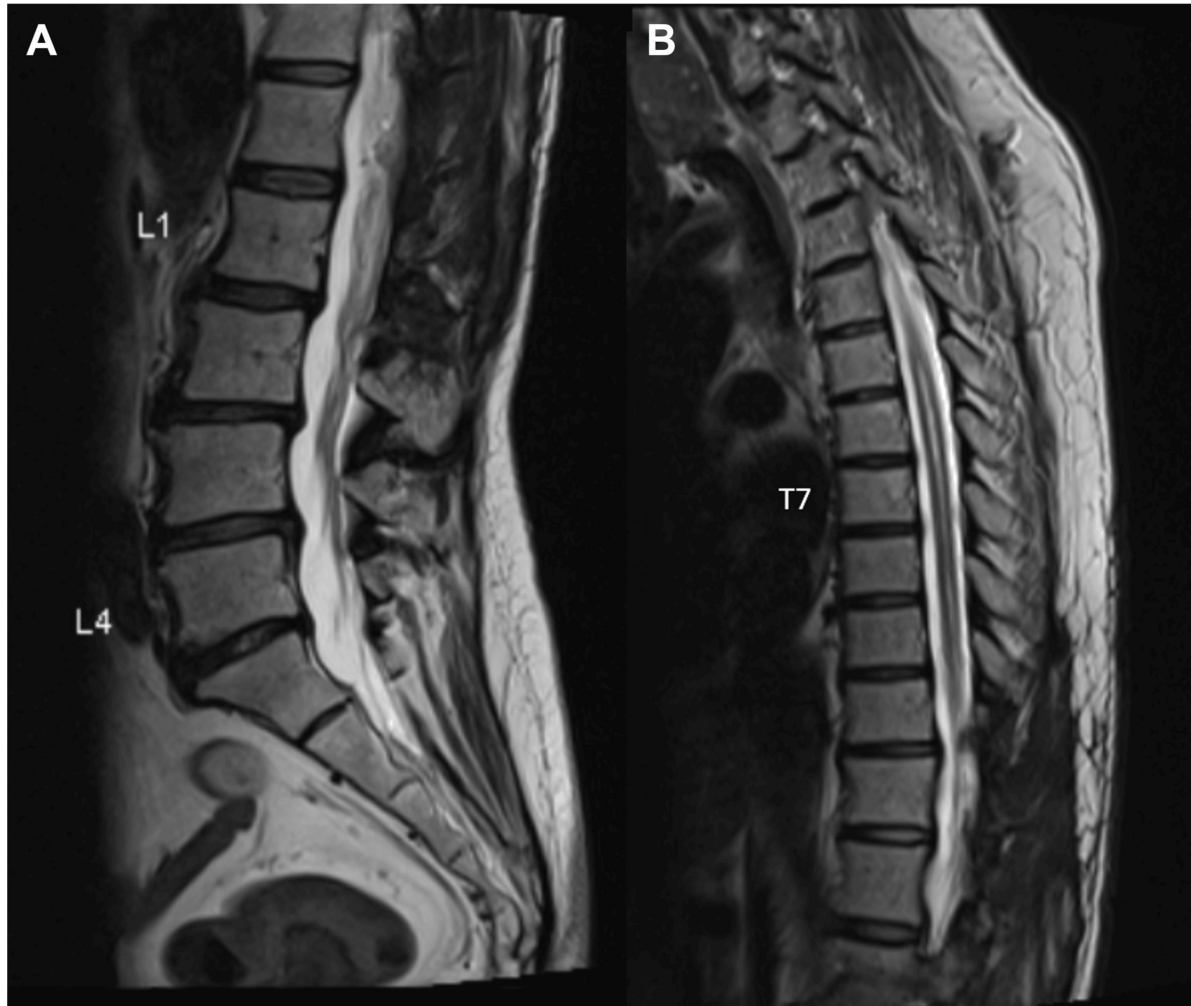
MRI of the spine performed six months after surgery T2-weighted imaging showed significant improvement of the known syrinx. T1-weighted imaging with contrast (not shown) showed a stable residual hemangioblastoma centered at T12 extending to T11 centrally. No new enhancing lesion.

Postoperatively, the patient’s left ankle dorsiflexion strength improved to 3/5 and remained stable at the six-month follow-up. MRI demonstrated a significant reduction of the syrinx and stability of the residual hemangioblastoma (Figure 8).

## Discussion

Spinal hemangioblastoma is a rare surgical condition. Preoperative imaging can aid in the diagnosis and guide subsequent management. MRI with gadolinium-based contrast is the investigation of choice for spinal hemangioblastoma [13]. Several characteristic MRI findings have been described in the literature.

In terms of the anatomic distribution, spinal hemangioblastomas predominantly localize to the thoracic (50%) and cervical (40%) regions of the spinal cord [2,7,18]. Most hemangioblastomas occur in the posterior region of the denticulate ligament [14,17,18], frequently abutting the dorsal root entry zone [14]. Approximately 66% of spinal hemangioblastomas arise at or near the spinal cord surface, either as intramedullary lesions extending beyond the cord’s surface or as exophytic tumors with the center external to the cord. Completely intramedullary tumors account for 25% of cases, and rarely, 8% present as intradural extramedullary lesions [7].

Generally, small hemangioblastomas (<10 mm) appear isointense relative to the spinal cord on T1-weighted imaging and hyperintense on T2-weighted imaging. Larger tumors tend to be hypointense or mixed hypo- and isointense on T1-weighted images and of heterogeneous intensity on T2-weighted images [7,19]. Contrast-enhanced MRI reveals well-demarcated, intense enhancement of the tumor nodule [7,19]. This pronounced enhancement is a hallmark feature, attributed to the high vascularity of the tumor parenchyma, which comprises thin-walled, closely packed blood vessels interspersed with stromal cells [19].

Vascular flow voids within or adjacent to the tumor may be seen on T2-weighted imaging, indicative of enlarged feeding or draining vessels. These flow voids are typically seen when the tumor is 25 mm or greater in size and are not associated with tumors less than 15 mm in size [19,20]. If present, they can serve as a distinguishing feature from other intramedullary neoplasms.

A significant proportion of spinal hemangioblastomas have been associated with syringomyelia, ranging from 50-100% across various studies [5,7,17-19,21-23]. There is no consensus on the mechanism of syrinx formation in spinal hemangioblastoma. Most authors believe it is due to transudation from tumor vessels and secretion by tumor cells, leading to elevated venous pressure in the spinal veins, and consequently the development of syringomyelia [17,19]. Alterations in blood perfusion and increased hydrostatic pressure throughout the spinal cord may also play a role [17]. A characteristic MRI feature of spinal hemangioblastoma is the large size of the syrinx relative to the size of the tumor [17,19]. Syrinx formation is not specific for spinal hemangioblastoma and can be seen with other spinal cord tumors, such as ependymoma and astrocytoma. However, radiologic findings of a small, superficial, intramedullary nodule with vivid enhancement and extensive syrinx formation are characteristics of spinal hemangioblastoma [7,19].

Another unique feature of hemangioblastoma is spinal cord enlargement extending beyond the tumor margins and distinct from a syrinx [7,20]. The cause of this spinal cord enlargement may be arteriovenous shunting, venous congestion, or an edema-promoting factor secreted by the tumor [24]. However, this is not present in all cases, and a small intramedullary hemangioblastoma may not induce significant cord enlargement or syrinx [7].

In this case, the MRI findings of a diffuse syrinx with associated cord expansion align with the typical imaging characteristics of spinal hemangioblastomas described in the literature. While contrast enhancement is a hallmark of these tumors due to their high vascularity, the enhancement in this case was heterogeneous, possibly due to the solid-cystic components. Intraoperatively, the highly vascular lesion and engorged venous complex are consistent with the known vascular characteristics of hemangioblastomas. The tumor’s location and interface with the spinal cord are typical for hemangioblastomas, which are commonly dorsolateral and arise at or near the spinal cord surface.

The MRI appearance of spinal hemangioblastomas necessitates differentiation from other intramedullary lesions, including ependymomas, astrocytomas, and cavernous malformations. While ependymomas and astrocytomas may also present with cord expansion and contrast enhancement, they generally lack the intense, homogeneous enhancement and associated flow voids characteristic of hemangioblastomas [3,20,25]. Cavernous malformations typically exhibit a “popcorn-like” appearance with mixed signal intensities due to hemorrhage and hemosiderin deposition, distinguishing them from the uniform enhancement pattern of hemangioblastomas [20,26]. A comprehensive evaluation for a primary tumor, such as renal carcinoma, is essential, particularly in patients with VHL disease [19].

Extramedullary hemangioblastoma is rare but can arise in the lumbar or sacral spinal canal, possibly in association with exiting nerve roots [7]. In this location, it may resemble meningioma or schwannoma. Distinguishing features that are more indicative of a hemangioblastoma include intense contrast enhancement and associated dilated vessels [7].

Preoperative recognition of hemangioblastoma affects management [7], including preoperative planning, consideration for angiography, indication for biopsy, preparation of blood products, and preoperative patient counselling.

While there are no established guidelines regarding the use of angiography for spinal hemangioblastomas, most authors agree that it is rarely necessary (and does not confer any additional benefit) [17,27]. Moreover, preoperative embolization is not routinely performed due to the inherent risks of the procedure and the high success rate of microsurgical resection [27]. While preoperative angiography and embolization may be performed in select cases for patients with very large hemangioblastomas [27,28], there is currently no evidence supporting their use [28,29]. Another adjunct is indocyanine green angiography, which can be used intraoperatively to assess the extent of resection and visualize associated vascular anatomy. However, its use has not impacted the extent of resection or complication rates [10].

The standard surgical strategy for spinal intramedullary tumors involves meticulous delineation of the tumor-cord interface. If a clear interface is absent or intraoperative frozen section suggests an anaplastic tumor, gross total resection should be abandoned [30]. However, in this case, biopsy was contraindicated due to the tumor’s vascularity, posing a high risk of hemorrhage.

Preoperative recognition of a hemangioblastoma would have facilitated better surgical planning, including the preparation of blood products to manage potential hemorrhage. It would also have guided preoperative patient counseling regarding bleeding risks and possible neurological deficits.

In this case, the diagnosis of hemangioblastoma was only suspected intraoperatively, and the surgery was challenging due to extensive bleeding and a drop in neuromonitoring signals. Fortunately, the patient’s neurological status improved postoperatively. The case was discussed at a multidisciplinary team meeting, where the options of repeat resection versus MRI surveillance were considered. Following an informed shared decision-making process with the patient, the decision was made for MRI monitoring. Subsequent imaging demonstrated a significant reduction of the syrinx.

## Conclusions

Spinal hemangioblastomas are rare, highly vascular lesions that require a high index of suspicion for diagnosis. The finding of one well-demarcated, superficially located, intramedullary, intensely enhancing nodule with extensive syrinx formation is highly suggestive of hemangioblastoma. Preoperative recognition of spinal hemangioblastoma can aid in surgical planning.
